# Current and future therapies to treat impaired awareness of hypoglycemia

**DOI:** 10.3389/fphar.2023.1271814

**Published:** 2023-10-24

**Authors:** Erica L. Macon, Micah H. Devore, Yu Kuei Lin, Megan B. Music, Mason Wooten, Colleen A. McMullen, Andrea M. Woodcox, Ashlee R. Marksbury, Zachary Beckner, Bansi V. Patel, Lily A. Schoeder, Ashley N. Iles, Simon J. Fisher

**Affiliations:** ^1^ Division of Endocrinology, Diabetes and Metabolism, Department of Internal Medicine, College of Medicine, University of Kentucky, Lexington, KY, United States; ^2^ Division of Metabolism, Endocrinology and Diabetes, Department of Internal Medicine, University of Michigan Medical School, Ann Arbor, MI, United States

**Keywords:** impaired awareness, hypoglycemia, unawareness, counterregulation, diabetes, insulin

## Abstract

In order to achieve optimal glycemic control, intensive insulin regimes are needed for individuals with Type 1 Diabetes (T1D) and insulin-dependent Type 2 Diabetes (T2D). Unfortunately, intensive glycemic control often results in insulin-induced hypoglycemia. Moreover, recurrent episodes of hypoglycemia result in both the loss of the characteristic warning symptoms associated with hypoglycemia and an attenuated counterregulatory hormone responses. The blunting of warning symptoms is known as impaired awareness of hypoglycemia (IAH). Together, IAH and the loss of the hormonal response is termed hypoglycemia associated autonomic failure (HAAF). IAH is prevalent in up to 25% in people with T1D and up to 10% in people with T2D. IAH and HAAF increase the risk of severe hypoglycemia 6-fold and 25-fold, respectively. To reduce this risk for severe hypoglycemia, multiple different therapeutic approaches are being explored that could improve awareness of hypoglycemia. Current therapies to improve awareness of hypoglycemia include patient education and psychoeducation, the use of novel glycemic control technology, pancreas/islet transplantation, and drug therapy. This review examines both existing therapies and potential therapies that are in pre-clinical testing. Novel treatments that improve awareness of hypoglycemia, via improving the counterregulatory hormone responses or improving hypoglycemic symptom recognition, would also shed light on the possible neurological mechanisms that lead to the development of IAH. To reduce the risk of severe hypoglycemia in people with diabetes, elucidating the mechanism behind IAH, as well as developing targeted therapies is currently an unmet need for those that suffer from IAH.

## Impaired awareness of hypoglycemia—complication of insulin treated diabetes

For people with diabetes, hypoglycemia is caused by excess insulin action in the setting of impaired counterregulation. In people who rely on insulin therapy to control their blood sugar levels, episodes of hypoglycemia increase the risk for subsequent episodes of hypoglycemia as part of a vicious cycle (Cryer, 1993; Davis et al., 2000; Muneer, 2021). With recurrent episodes of hypoglycemia, brain glucose sensing becomes impaired and the usual neuronal signaling pathways that elicit a counterregulatory response to raise blood glucose levels are diminished (Muneer, 2021). Thus, in the setting of impaired insulin and glucagon responses to hypoglycemia, recurrent hypoglycemia induces a syndrome of Hypoglycemia Associated Autonomic Failure (HAAF) that is composed of an impaired awareness of hypoglycemia (IAH) and a blunted counterregulatory response (Davis et al., 2000). The blunted counterregulatory response consists of impaired adrenergic signaling that results in an impaired endogenous epinephrine secretion from the adrenal medulla (Muneer, 2021). In conjunction with reduced autonomic signaling, neurogenic symptoms of hypoglycemia are also attenuated. Thus, people with recurrent episodes of insulin-induced hypoglycemia have a diminished ability to detect hunger, sweating, tremors, or other signals that indicate that carbohydrates should be ingested to raise blood glucose levels (Cryer, 1993; Davis et al., 2000; Muneer, 2021).

With better glycemic control, patients with Type 1 (T1D) and insulin-dependent Type 2 Diabetes (T2D) have been able to reduce the risk for diabetes complications (e.g., retinopathy, neuropathy, and nephropathy) (Holman et al., 2008; Diabetes, 2016). Yet, as patients intensify glycemic control, the risk for iatrogenic hypoglycemia increases proportionately (Holman et al., 2008; Lipska et al., 2014; Diabetes, 2016; Chittineni et al., 2019). From 1999 to 2011, there has been a trend in reduced hospitalizations for hyperglycemia, but the rates of hospital admissions for severe hypoglycemia remain almost two-fold higher than those for hyperglycemia (Lipska et al., 2014). Severe hypoglycemia is therefore a burden for patients with established diabetes and increases the risk of adverse clinical outcomes (Mantovani et al., 2016). Severe hypoglycemia is also associated with impaired cognitive function (Deary et al., 1993). Overall, hypoglycemia remains the rate-limiting factor in patients striving to achieve optimal glycemic control in people with Type 1 and longstanding Type 2 Diabetes (Cryer, 2014).

In addition to recurrent episodes of hypoglycemia, other factors can impair counterregulation and/or induce IAH and thus contribute to the risk for hypoglycemia (Martyn-Nemeth et al., 2019). Nocturnal hypoglycemia is also prevalent in T1D. Barnard et al. showed that 35% of patients with T1D self-reported having experienced hypoglycemia while sleeping (Barnard et al., 2016). People with IAH often fail to wake from sleep to correct an episode of hypoglycemia due to their impaired activation of the autonomic nervous system in response to hypoglycemia (Jones et al., 1998; Banarer and Cryer, 2003). Another confounder in achieving optimal glycemic control is exercise (Martyn-Nemeth et al., 2019; Romeres et al., 2021). An bout of exercise increases glucose utilization and also increases tissue sensitivity to insulin. This combination lowers blood glucose and increases the risk and incidence of hypoglycemia, compared to insulin alone (Munoz et al., 2018; Nguyen et al., 2021; Romeres et al., 2021). Moreover, antecedent exercise has been shown to blunt awareness and the counterregulatory response to hypoglycemia, thus contributing to the development of HAAF (Galassetti et al., 2001; Sandoval et al., 2004).

## Diagnosis of impaired awareness of hypoglycemia

Since HAAF increases the risk for severe hypoglycemia by 25-fold (Cryer, 2016), it is important for healthcare providers to determine if their patients can sense hypoglycemia. Several questionnaires have been developed to assist the diagnosis of IAH. The Gold Score is a hypoglycemia questionnaire in which subjects are asked a single question, “Do you know when your hypoglycemic episodes are commencing?” (Gold et al., 1994; Ang et al., 2023). The patient responds using a 7-point Likert scale where one to two denotes awareness, 3 is equivocal, and four to seven indicates unawareness (Gold et al., 1994). The Clark Score is a more multi-dimensional survey which consists of eight questions that are used to achieve objective answers regarding awareness of hypoglycemia (Clarke et al., 1995; Ang et al., 2023). With a score range from 0 to 7, a response total of 4 or above indicates IAH (Clarke et al., 1995; Lin et al., 2019; Ang et al., 2023). The Pedersen-Bjergaard questionnaire asks patients to recall their previous experiences with hypoglycemia and asses their ability to recognize symptoms of hypoglycemia (Pedersen-Bjergaard et al., 2003). The Pedersen-Bjergaard Score provide a unique understanding of the multiple levels of awareness ranging from “normal awareness, impaired awareness, and unawareness” (Pedersen-Bjergaard et al., 2003). HypoA-Q is a more recently developed hypoglycemia assessment instrument that is used to characterize IAH and allow for “a more definitive diagnosis of IAH” (Speight et al., 2016). Since the IAH questionnaires vary, some discrepancies can arise such as overestimating impaired awareness in populations that may still have awareness intact, thus leading to the apparent failure of some studies to detect significant improvements in response to clinical interventions (Sepulveda et al., 2020; Ghandi et al., 2021; Ang et al., 2023).

These questionnaires have been criticized for 1) having a high degree of inter-questionnaire variability in identifying subjects with IAH and subjects with impaired counterregulation, 2) susceptibility to recall bias by the subject, 3) lacking sensitivity to detect changes in hypoglycemia awareness over a short period, and 4) were developed in the pre-continuous glucose monitor (CGM) era (excluding HypoA-Q). Also, hypoglycemia questionnaires do not distinguish whether awareness reflects true restoration of hypoglycemia awareness (i.e., improvement in symptom recognition) *versus* or “electronic awareness” by noting the glucose trace falling or hearing the alarms with CGM (Reddy et al., 2018; Lin et al., 2020a).

Hypoglycemic questionnaires do have many meritorious qualities in that they are 1) inexpensive, 2) non-invasive, and 3) amenable to out-patient settings. In addition, these questionnaires have been validated and adapted to populations beyond their original demographic (Alkhatatbeh et al., 2019; Yosef, 2021; Takagi et al., 2022a). Added benefits for these questionnaires include them being flexible to meet a large sample size (Sepulveda et al., 2020; Ghandi et al., 2021; Takagi et al., 2022a; Ang et al., 2023). More recent studies also demonstrate that patients with IAH diagnosed by questionnaires continue to experience higher risks of severe hypoglycemia (Lin et al., 2019; Lin et al., 2022).

## Impaired awareness of hypoglycemia (IAH) therapies

Mistimed or imprecise dosing of insulin increases the likelihood of hypoglycemic events and recurrent episodes of hypoglycemia lead to the development of IAH (Cryer, 1993; Davis et al., 2000; Geddes et al., 2008; van Meijel et al., 2020; Muneer, 2021). In addition to people who have a history of hypoglycemic events, certain populations are at a greater risk for hypoglycemic episodes and IAH, such as the young, elderly, and those with comorbidities (Munshi et al., 2011; Cengiz et al., 2013; Farrell and McCrimmon, 2021). Thus, identifying individuals who are at a higher risk for severe hypoglycemia and IAH is a priority for clinical providers and their patients in order to decrease the incidence of both events.

In spite of their limitations (see above), the most practical method to assess for IAH in a clinical setting is hypoglycemia questionnaires. However, if patients are not asked about hypoglycemia or fail to report asymptomatic hypoglycemia, the diagnosis of IAH can be missed (Farrell and McCrimmon, 2021). Therefore, it is extremely important for providers to inquire about and for patients to be educated about IAH. After identification of IAH, the goals would be to provide at-risk patients with strategies to recognize and avoid hypoglycemia.

Prior to advanced diabetes technology such as CGMs and the automated insulin delivery systems, several of these earlier studies demonstrated that the scrupulous avoidance of recurrent episodes of hypoglycemia could restore (at least partially) awareness of hypoglycemia (Cranston et al., 1994a; Fanelli et al., 1996; Fritsche et al., 2001). To the extent that HAAF may be reversed (at least partially), avoidance of hypoglycemia is a practical goal treatment for IAH. Unfortunately, even with modern technology, complete avoidance of hypoglycemia is difficult, compounded by the evidence that only one to two episodes of hypoglycemia are sufficient to induce IAH (Galassetti et al., 2001). In the setting of intensive glycemic control achieved with intensive insulin delivery, complete avoidance of hypoglycemia may not be realistic for some individuals. The question remains whether complete avoidance of hypoglycemia using the latest strategies can restore hypoglycemia awareness. Conversely, if iatrogenic hypoglycemia cannot be completely avoided, analysis of CGM data will enable researchers to determine the maximal amount of time spent in the hypoglycemic range that will still allow for amelioration/restoration of IAH and the defective counterregulatory response.

**FIGURE 1:**
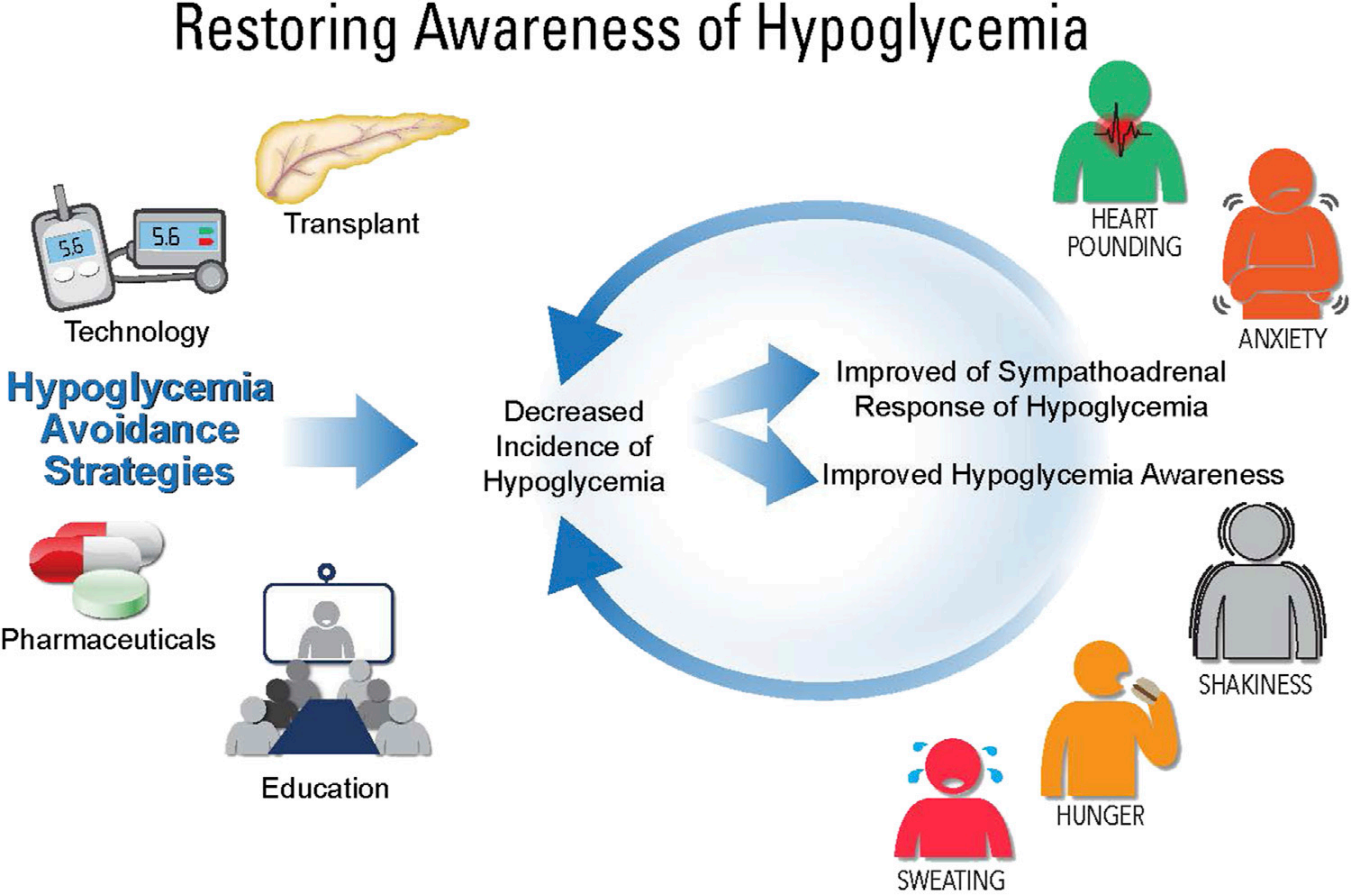
Restoring awareness of hypoglycemia. While there is no direct treatment for impaired awareness of hypoglycemia (IAH), there are therapies that can help avoid hypoglycemia, which include: education, pharmaceuticals, technology, and transplantation (whole pancreas or islet cell). Using these therapies, hypoglycemia can be avoided leading to improve sympathoadrenal responses of hypoglycemia and awareness of hypoglycemia.

Given the complexity of IAH, a variety of clinical treatment considerations have been investigated to decrease hypoglycemia and the cycle of IAH (Figure 1). In the following sections, various treatment options for IAH will be discussed (see Table 1).

**TABLE 1:** Clinical therapies for impaired awareness of hypoglycemia (IAH).

Clinical therapies				
Type	Sub-type	Description/Purpose	Outcome	Citation(s)
Education				
	HARPdoc Program	6-week group program with motivational interviewing and cognitive behavioral theory aiming to reduce negative cognitions around IAH	Improved mental health of participants, decreased severe hypoglycemic episodes similar to BGAT	de Zoysa et al. (2014), Amiel, 2022; Amiel et al. (2022), Jacob et al. (2022b)
	BGAT	8-week psychoeducational training program to anticipate, prevent, and treat extreme BG levels	Repeatedly efficacious in multicenter trials, interactive/personalized unit materials	Gonder-Frederick et al. (1997), Gonder-Frederick et al. (2000), Cox et al. (2001), Cox et al. (2004), Snoek et al. (2008), Soukup et al. (2019)
	DAFNE-HART	Program centered around problematic hypoglycemia, specifically individual’s motivational and cognitive barriers	Training to address cognitive/motivational barriers to improving hypoglycemia	de Zoysa et al. (2014)
	HyPOS	Five, 90-min lesson over 5-week of educational material to treat patients with hypoglycemia complications	Training centered around improving awareness and decreasing hypoglycemia	Hermanns et al. (2007), Hermanns et al. (2010)
	HypoAware	Three, 2.5 h group sessions over 4-week +2 online modules and aimed to improve hypoglycemic symptom recognition, risk awareness, prevention of hypoglycemia, and coping with hypoglycemia	Can be used in both T1D and severe T2D patients and evaluated in multiple centers	Rondags et al. (2016)
Technology				
	Glucometers	Manual, handheld device that determines blood glucose in real-time	Multiple models on the market, extensively researched, affordable	Khan et al. (2006), Sonmez et al. (2010), Bellary et al. (2012), Clarke and Foster (2012), Francescato et al. (2012), Freckmann et al. (2012), Mishra et al. (2022), Ebekozien and Shah (2023)
	CGM	Continuous glucose monitoring technology generates data for better management of diabetes, low glucose alarms and alerts	Decrease hypoglycemia events and severity, has alarms to alert subjects with IAH to a hypoglycemic event	Cryer (2013), Shivers et al. (2013), Rickels et al. (2016), Agiostratidou et al. (2017), Rickels et al. (2018), Battelino et al. (2019), Cook et al. (2019), Henriksen et al. (2019), Advani (2020), Battelino and Bergenstal (2020), Lin et al. (2020c), Kalra et al. (2021), Cobry et al. (2022), Perez Cavero et al. (2022), Renard et al. (2022), Vieira et al. (2022), Lin et al. (2023a), DeSalvo et al. (2023), Dovc et al. (2023)
	Closed Loop Systems	CGM is combined with a sensor augmented insulin pump that automates insulin delivery	Decrease the episodes and fear of hypoglycemia	Kudva et al. (2021), Takagi et al. (2022b)
	Sensor Augmented Pumps	Also known as an “open-loop system”—SAPs are insulin pumps that communicate with CGMs for optimal glucose management	Improved awareness via Clarke assessment	Takagi et al. (2022b)
	Automated Insulin Delivery	CGM combined with a partially automated insulin delivery system with user input for insulin meal boluses	Improved awareness (with robust measurements) and counterregulatory responses	Burckhardt et al. (2021), Malone et al. (2021), Flatt et al. (2023), Nattero-Chávez et al. (2023)
Transplantation				
	Pancreas	Transplantation of tissue/cells to restore endogenous insulin and glucagon secretion/action	Fully restore hypoglycemia awareness	Kendall et al. (1997)
	Islet Cell			Rickels et al. (2015), Rickels et al. (2016), Rickels et al. (2022), Hu et al. (2023)

Abbreviations: continuous glucose monitor (CGM), blood glucose (BG), impaired awareness of hypoglycemia (IAH), severe hypoglycemia (SH), blood glucose awareness training (BGAT), dose adjustment for normal eating (DAFNE), hypoglycemia awareness restoration training (HART), hypoglycemia, anticipation, awareness, and treatment training (HAATT), hypoglycemia (HYPO), gastrointestinal (GI), Type 1 Diabetic (T1D), Type 2 Diabetic (T2D), Automated Insulin Delivery (AID), Close Loop Systems (CLC).

Strategies to avoid hypoglycemia include transplantation (pancreas or islet cells), technology (e.g., CGM, insulin pumps, hybrid closed loop), pharmaceuticals, and patient education. The overarching goal is to decrease incidences of hypoglycemia and thereby restore both awareness of hypoglycemia and improve the counterregulatory response to hypoglycemia.

### Education and psychoeducation

Fundamentally, the most pressing issue with IAH is the inability to sense when blood glucose concentrations fall to severe levels (i.e., requiring assistance from another individual in order to treat the episode of hypoglycemia). Diabetes education programs have been successfully employed to improve glycemic control and the overall health of people with T1D and T2D (Siminerio et al., 2018). Although not specifically designed to treat IAH, some of the original educational programs that focused on glycemic management resulted in improving hypoglycemia awareness. The Diabetes Teaching and Treatment Program (DTTP) demonstrated (in a 12-year follow-up) that the rates of hypoglycemia were reduced and the improvement in HbA1c was sustained after attending educational programs (Plank et al., 2004; Samann et al., 2005). Modeled after DTTP, the dose adjustment for normal eating (DAFNE) training program showed in a 1-year follow up that subjects had improved awareness of hypoglycemia and reduced rates of severe hypoglycemia (Group, 2002; Hopkins et al., 2012). The Tayside Insulin Management education program also showed reduced rates of severe hypoglycemia, reduction in HbA1c, and a 25% improvement in awareness after 6-month of the program (Jordan et al., 2013).

Given the increased risk of hypoglycemia with intensive glycemic control, educational programs began to focus on improving awareness of hypoglycemia. More specific psychological training and bio-psycho-behavioral techniques have been shown to help people with diabetes improve their awareness. The Blood Glucose Awareness Training Program (BGAT) is an IAH focused psychoeducational program (Cox et al., 2004). BGAT equips people with T1D with comprehensive and practical content including insulin, dietary, physical activity, blood glucose management, and most importantly, its goal is to heighten an individual’s ability to detect and avoid hypoglycemia. Since its inception, BGAT has undergone several revisions as a result of multicenter trials across the globe. BGAT is available outside of a clinical setting, which enables it to reach more people and decrease the workload in the clinic (Cox et al., 2006). While still extremely effective at improving overall blood glucose awareness, BGAT did not intentionally set out to assess IAH. Nonetheless, several studies demonstrated the ability of BGAT in improving hypoglycemia awareness (Cox et al., 1995; Snoek et al., 2008); including a recent trial with exclusively IAH subjects that had recurrent severe hypoglycemic episodes (Jacob et al., 2022b).

After the success of BGAT, educational programs entered an era of utilizing hypoglycemia symptom detection training for improving/treating hypoglycemia awareness/IAH. Adapted from BGAT, the HypoAware training program focused on training and empowering people with T1D and advanced T2D to reduce episodes of hypoglycemia, improve awareness, and reduce fear of hypoglycemia (Rondags et al., 2016). Another educational program for treating diabetic patients with hypoglycemia problems (HyPOS), focused on optimizing intensive insulin therapy. Additional dependent variables were assessed then in the previous tests including hypoglycemia detection, hypoglycemia treatment, quality of life, and rates of mild/severe/very severe hypoglycemia (Hermanns et al., 2007). In comparison to BGAT, the HyPOS study found a 41% improvement (BGAT 24%) in hypoglycemia detection and an 18% reduction in mild hypoglycemia (BGAT 12.5%) (Hermanns et al., 2007). Additionally, the long-term benefits of HyPOS curriculum remained after a 31-month follow-up (Hermanns et al., 2010). Similar to the HypoAware adaptation from BGAT, the dose adjustment for normal eating (DAFNE)—Hypoglycemia Awareness Restoration Training (HART) was developed from the DAFNE program. DAFNE-HART Researchers hypothesized that the IAH persistence seen in the DAFNE project was due to maladaptive beliefs and/or motivational barriers. The DAFNE-HART in a pre-post trial with 23 participants demonstrated that psychology plays an important in the development of IAH. Of note, 45% of subjects improved their awareness and 85% of subjects experienced no further episodes of severe hypoglycemia in a 12-month follow-up (de Zoysa et al., 2014).

Building on the DAFNE-HART program, the Hypoglycemia Awareness Restoration Programme for People with Type 1 Diabetes and Problematic hypoglycemia Persisting despite optimized self-care (HARPdoc) was developed as a multidisciplinary strategy targeting cognitive in subjects with IAH. The HARPdoc program was recently evaluated and compared its effectiveness with BGAT in a population who continued to have IAH and developed recurrent severe hypoglycemia despite prior structured diabetes education and offered advanced diabetes technologies (Jacob et al., 2022b). HARPdoc and BGAT were similarly able to improve awareness of hypoglycemia and decrease the rate and fear of hypoglycemia (Jacob et al., 2022b). HARPdoc was also shown to decrease maladaptive hypoglycemia beliefs, diabetes distress and depression and anxiety symptoms which was not demonstrated in recipients of BGAT (Jacob et al., 2022b). HARPdoc brain responses have also been compared to the HypoAware study (Jacob et al., 2022a). While limited in statistical power (only compared 12 subjects), HARPdoc was able to determine awareness status more accurately during two-stepped hyperinsulinemic-hypoglycemic clamps (Jacob et al., 2022a). In comparison to HypoAware, the HARPdoc treatment showed that the superior frontal gyrus region was more activated during hypoglycemia, indicating improved self-awareness and symptoms associated with hypoglycemia (Jacob et al., 2022a).

Treatment of IAH in people with T2D has been studied to a much lesser extent compared to studies in people with T1D. The Common Sense Model (CSM) assessed illness perceptions in subjects with T2D and IAH on insulin therapy (Shen et al., 2022). While the study showed that the overall welfare and coping of subjects was improved, CSM did not change fear or awareness of hypoglycemia (Shen et al., 2022). These results may be due to a short-duration of follow-up (1 and 3-month).

The efficacy of educational programs cannot be understated. Educational programs that use close and frequent patient contact (Cranston et al., 1994b; Fanelli et al., 1994; Little et al., 2014) have a clinical benefit that may be larger than the beneficial effect observed with diabetes technological interventions (*vide infra*). For example, the HypoCOMPaSS trial (Comparison of Optimized MDI *versus* Pumps with or without sensors in severe hypoglycemia) (Cox et al., 2006) demonstrated improvements in hypoglycemia awareness and reduction in severe hypoglycemia with intensive hypoglycemia-focused education and close monitoring program, with non-differential effects between groups using more traditional or advanced glucose monitoring and insulin administration technologies (Yeoh et al., 2015). The positive effects of the HypoCOMPaSS program were maintained at least 2 years after the completion of the original study (Speight et al., 2019).

### Technology

For people with IAH, hypoglycemia is often detected not by symptoms, but with glucose monitoring technology (e.g., handheld glucometers, continuous glucose monitors, low glucose alerts/alarms, etc.). Unquestionably, diabetes technologies have markedly improved treatment for people with diabetes (Akturk and Garg, 2019). It is indeed unfortunate that the more widespread use of these valuable technologies is limited by socioeconomic inequalities (Bellary et al., 2012; Scott et al., 2017; Alcantara-Aragon, 2019; Fauzi et al., 2022; Mishra et al., 2022). Novel diabetes technologies for assessing glucose levels can be classified (broadly) into three tiers; 1) CGM (or flash/intermittent monitors), 2) CGM with sensor augmented insulin pump, and 3) automated insulin delivery systems (Ebekozien and Shah, 2023). Although these technological advances have unquestionably helped to improve glycemic control and reduce that incidence of severe hypoglycemia in people with T1D, the extent to which these technologies can restore awareness of hypoglycemia remains an active area of investigation (Choudhary et al., 2013; Little et al., 2014; van Beers et al., 2016; Heinemann et al., 2018; Rickels et al., 2018; Cook et al., 2019; Lin et al., 2019; Lin et al., 2020b; Pratley et al., 2020; Sepulveda et al., 2020; Burckhardt et al., 2021; Bahrami et al., 2022; Takagi et al., 2022b).

#### Continuous glucose monitors (CGM)

CGMs have revolutionized diabetes management. Since CGMs can measure glucose every 5 min and alert patients of impending low (as well as high) glucose levels, they represent a major leap forward in glycemic management over handheld glucometers. Recent data has shown that CGM users (N = 5,506/11,469) had better glycemic control (lower median HbA1C, 7.7%) and lower rates of severe hypoglycemia compared to non-CGM users (DeSalvo et al., 2023). While HbA1c has been the gold standard for assessing long-term glycemic control, the data available from CGMs are making these devices the new standard of care (Battelino et al., 2019; Advani, 2020; Battelino and Bergenstal, 2020; Kalra et al., 2021; Dovc et al., 2023). CGMs indicate the amount of time subjects experience hypoglycemia and how often these episodes go unnoticed. Henriksen et al. evaluated 153 men with T1D and found that 87% had at least one hypoglycemic episode per day (Henriksen et al., 2019). Additionally, they noted that of all the hypoglycemic events captured by the CGMs (≤54 and <39.6 mg/dL), ∼74% of them were asymptomatic (Henriksen et al., 2019). This study highlighted the persistent prevalence of IAH in people with T1D despite CGM usage.

The Advanced Technologies and Treatments for Diabetes Congress formed a panel of expert individuals to compose CGM guidelines for clinician use (Battelino et al., 2019). These guidelines include: the number of days CGM was worn, percentage (%) of CGM active, mean glucose, glucose management indicator, glycemic variability, time above range (TAR), time in range (TIR), and time below range (TBR) (Battelino et al., 2019). To determine if these metrics would be useful in identifying individuals with IAH, Lin et al. showed that half of the subjects with IAH met the proposed guidelines for hypoglycemia (Lin et al., 2020c). More specifically, the % of TBR (<70 and <54 mg/dL) and hypoglycemic events that lasted 15 or 20-min provide both acute and chronic glycemic history, respectively (Lin et al., 2020c). Additionally, using CGM data, researchers proposed a new CGM metric to identify IAH. One study assessed intermittent CGM use to identify risk factors for IAH and glycemic patterns (Vieira et al., 2022). After analyzing CGM data it was proposed that the duration of a hypoglycemic episode (≥106.5 min) was one criterion by which IAH could be identified (Vieira et al., 2022).

While CGM usage reduces the incidence and severity of hypoglycemic episodes, there are conflicting reports as to whether CGM usage results in an improved awareness of hypoglycemia. A recent study (Ali et al., 2023) showed that using a CGM decreased IAH by 50% compared to previous years in individuals with T1D. In a larger population (N = 90 subjects) T1D subjects who spent greater than 1.5 h/day in hypoglycemia were given the Eversense^©^ (Ascensia Diabetes Care, United States) CGM to determine if it decreased the time spent in hypoglycemia. Researchers found that after 3–4 months subjects decreased their TBR, which was associated with increased TIR and was sustained after 5–6 months (Renard et al., 2022); however, hypoglycemia awareness status was not assessed in the trial. Decreased TBR could improve awareness; however, this study found (Renard et al., 2022) no improvement in HbA1c after 6-month indicating that glycemic control was still not attained.

While CGM technology has made patients and clinicians more cognizant of the frequency of hypoglycemic events, it is clear that GCM use does not eliminate hypoglycemic episodes (Lin et al., 2023a; Hu et al., 2023). Even a long-term study (18-month of CGM use) failed to improve both symptomatic responses to hypoglycemia and hormonal counterregulatory responses (Rickels et al., 2018). Consistent with these disheartening findings, our research team has consistently found a persistently high prevalence of IAH among CGM users, again dispelling any notion that CGM usage somehow restores awareness of hypoglycemia (Lin et al., 2019; Lin et al., 2020a; Lin et al., 2020b; Lin et al., 2020c; Lin et al., 2022).

Despite the use of a CGM, the reasons for only partial improvements in HAAF remain largely unknown, but have been attributed to 1) failure to adequately/scrupulously avoid recurrent hypoglycemia for a sufficiently long duration, 2) a methodology issue wherein self-reported hypoglycemia awareness questionnaires may lack sufficient sensitivity to note an improvement in hypoglycemia awareness in follow up testing, and 3) heterogeneous factors distinct from recurrent hypoglycemia (e.g., age, duration of diabetes, glycemic variability) that may play a pathophysiological role the development/perpetuation of IAH, and 4) the mistrust of CGM glucose information during asymptomatic episodes and other barriers to hypoglycemia self-management (Lin et al., 2023b) which further perpetuate future hypoglycemic episodes. Alternatively, ineffective use of CGM hypoglycemia-informing features (Lin et al., 2023a), alarm fatigue, psychosocial/behavioral factors (Shivers et al., 2013; Cook et al., 2019; Cobry et al., 2022; Lin et al., 2023a), and/or other factors not related to hypoglycemia avoidance may play an important role in this apparent failure to completely restore both counterregulation and awareness in subjects with HAAF. Identifying these and other factors that might be necessary for the restoration of hypoglycemia awareness are needed to develop mitigation strategies and achieve an overall goal of reducing the burden of disease in people with T1D.

#### Closed-loop systems (CLS)

In addition to CGMs, people with diabetes also use insulin pump delivery systems (thus replacing multiple daily injections of insulin). The combination of CGM and insulin pump technologies have been described as the holy grail of diabetes management (Templer, 2022). The CLS was developed by people with T1D and their families by creating an open-source software (Templer, 2022). This software connects CGMs and insulin pumps to a software through a phone or computer, and analyzes blood glucose to make decisions that adjust insulin delivery (Templer, 2022). Currently, there are three available platforms that combine a CGM and insulin pump: Loop, OpenAPS (Open Source Artificial Pancreas), and AndroidAPS (Android Artificial Pancreas). However, as of yet, none of these platforms have been approved by the Federal Drug Administration (Palmer et al., 2021). Given the self-service (“DIY”) nature of a fully CLS, it has been difficult to assess their usefulness on IAH until the International Diabetes Closed-Loop Trial (Kudva et al., 2021). In this trial, subjects were randomized into a CLS or a sensor augmented pump (SAP). Hypoglycemia fear (Cox et al., 1987; Irvine et al., 1994), diabetes distress scale (Polonsky et al., 2005), hypoglycemia awareness, hypoglycemia confidence, and hyperglycemia avoidance were assessed at baseline, three, and 6-months post-technology implementation. CLS subjects had improved hypoglycemia fear scores (at 6 months) and a tendency for improved confidence in managing hypoglycemia; however, awareness was not different between the technologies (Kudva et al., 2021).

#### Sensor augmented pumps (SAP)

With low (or predicted low) glucose values detected by CGM, sensor augmented pumps (SAP) allow for automated insulin suspension. By temporarily suspending insulin delivery, SAP can avoid (or limit) the severity of hypoglycemia (Steineck et al., 2017). SAPs have been shown to be useful in people with severe hypoglycemia (Ly et al., 2013; Maahs et al., 2014; Bosi et al., 2019) and have improved hypoglycemia awareness (Quirós et al., 2016; Sakane et al., 2023). One such study investigating T1D subjects with IAH (based on the Clarke questionnaire) were assessed at baseline and 3 and 6-month follow-ups after SAP + CGM implementation. The study reported a decrease in Hb1Ac, TAR, and Clarke scores; however, there was no change in TBR (Takagi et al., 2022b). Thus, authors concluded that the SAP improved glycemic control by decreasing hyperglycemia and may improve awareness; but counterintuitively, not by reducing TBR (Takagi et al., 2022b). Given both 1) the limited evidence of improvement in awareness with SAPs, and 2) the rapid commercialization of automated insulin delivery systems, IAH research has evolved to be conducted with the next level of technology, automated insulin delivery systems.

#### Automated insulin delivery systems

Automated insulin delivery (AID) systems use an algorithm to automate insulin delivery to manage sugar levels; however, it requires the user to manually enter meal insulin boluses and thus is often termed a “hybrid closed loop” rather than a fully “closed loop” (Templer, 2022). AID systems have been shown to be effective in both T1D adults and adolescents in improving HbA1c, increasing TIR, and decreasing hypoglycemia (Kovatchev et al., 2014; Bergenstal et al., 2016; Garg et al., 2017; ForlenzaGregory et al., 2019; Pulkkinen et al., 2023). Malone et al. (2021) examined the long-term benefit (18-month) on awareness using an AID in T1D subjects (Malone et al., 2021). No statistical improvement for awareness was found; but there was a trend in improvement from baseline (Malone et al., 2021). Burckhardt *et a*l. (2021) examined both arms of HAAF that perpetuate IAH, counterregulation and awareness (Burckhardt et al., 2021). While counterregulatory responses did not change (epinephrine, norepinephrine, cortisol, growth hormone) with the use of AID, the total symptom scores assessed (both adrenergic and neuroglycopenic) during a hypoglycemic clamp improved from baseline compared to subjects using a SAP alone (Burckhardt et al., 2021). In contrast to the Burckhardt study, Flatt et al. (2023), found that both awareness and counterregulatory response improved after the implementation of AID (although this study lacked a control group) (Hu et al., 2023). Another study examined CGM metrics and awareness after the implementation of an advanced AID, MiniMed 780G™ (Medtronic, Dublin, Ireland): multiple daily insulin system (Nattero-Chávez et al., 2023). Out of the 46 patients included in the study, 12 patients (27%) had IAH at the baseline screen based on Clarke scores. Regardless of previous technology, subjects with IAH had improved HbA1c and Clarke scores; however, authors included subjects with Clarke scores ≥3. A score of 3 on the Clarke score is borderline for IAH; therefore, some aware subjects could have been included in the statistical analysis in the described study (Nattero-Chávez et al., 2023). Additionally, diabetes education provided to the AID subjects could have, independently, played a role in improving awareness scores (Nattero-Chávez et al., 2023). The benefits of automated insulin delivery cannot be minimized; the aforementioned studies showed improvements in glycemic management and awareness.

It is worthwhile to note that while some intervention studies do demonstrate an improvement in hypoglycemia questionnaire scores, it is unclear if a statistical improvement is clinically relevant as study subjects often demonstrate a persistent impaired awareness of hypoglycemia (Burckhardt et al., 2021; Takagi et al., 2022b).

It should be noted that the study design is another factor contributing to these seemingly discordant results viz-a-viz the ability of technology to restore awareness of hypoglycemia. The putative factors that contribute to the short-term blunting of the sympathoadrenal response to hypoglycemia induced by a few bouts of antecedent hypoglycemia in non-diabetic subjects are almost certainly different from the factors that contribute to HAAF (having developed over years in people with T1D). Disparate patient inclusion criteria are also confounding factors when comparing results from different studies. For example, early studies that established the efficacy of hypoglycemia avoidance to improve autonomic symptom responses were conducted in subjects with relatively short disease duration (∼7 years) (Dagogo-Jack et al., 1994). Some of the more recent studies that fail to reproduce such marked improvements recruited subjects with longer disease (≥15 years) duration and (apparently) a particularly immutable impaired awareness (Yeoh et al., 2015; Iqbal and Heller, 2018). These and other factors may explain the apparent efficacy of early studies showing benefits with short term (one to three months) interventions in small cohorts (6–12 subjects with T1D). In contrast, recent interventions using the latest diabetes technologies failed to demonstrate an improvement in hypoglycemia awareness in larger cohorts (Pratley et al., 2020) and failed to improve autonomic symptom scores following a long-term (18-month) intervention (Rickels et al., 2018).

An alternative notion to the exclusively glucocentric etiology of HAAF, is the possibility that HAAF is a heterogeneous clinical entity that develops, in part due to recurrent hypoglycemia, but also develops due to other factors (e.g., long-standing diabetes, aging, glycemic variability, sleep, antecedent exercise, alcohol, autonomic neuropathy and/or changes in CNS metabolism and function) (Lin et al., 2020a). If these heterogeneous factors are indeed major factors that contribute to HAAF, then perhaps the failure to restore awareness of hypoglycemia with novel diabetes therapeutics (*vide supra*) is not necessarily due to a failure to scrupulously avoid recurrent hypoglycemia. Consequently, it is possible that multiple interventions addressing these many potential confounding variables may be necessary to completely restore normal awareness and counterregulation in all subjects.

### Transplantation (islet cell and pancreas)

For people with intractable episodes of severe hypoglycemia, whole pancreas or islet cell transplantation remains an important treatment option recommended by the American Diabetes Association (Robertson et al., 2004). Previous studies have shown both whole pancreas and islet cell transplantation are effective (almost immediately) at restoring endogenous insulin and glucagon secretion (Kendall et al., 1997; Rickels et al., 2015; Rickels et al., 2016). Sympathoadrenal responses and hypoglycemia associated symptoms may take >6 months to recover (Kendall et al., 1997; Rickels et al., 2015). More recent data from the Clinical Islet Transplantation Consortium (CIT-08 Study) showed that greater than 90% of subjects with islet-only or islet-after-kidney transplant were free of hypoglycemia (Rickels et al., 2022). The authors concluded that either treatment would be most appropriate for patients with IAH (Rickels et al., 2022). Virtual elimination of hypoglycemia with intrahepatic islet transplantation in subjects with T1D leads to improvement in hypoglycemia symptom recognition (Rickels et al., 2016). Following transplant, epinephrine response to hypoglycemia was improved at 6- months and normalized at 18- months and the symptoms of hypoglycemia were normalized at both time-points after transplant (Rickels et al., 2016). Supporting the glucocentric cause of HAAF, findings in transplant patients indicate that the prolonged, near complete elimination of hypoglycemia, can completely reverse HAAF. Transplantation is therefore a very effective treatment for IAH; however, like any tissue transplant program, both availability of tissues/cells, as well as transplant rejection, are the primary challenges with such approaches (Aggarwal et al., 2022; Opara et al., 2023).

### Pharmacological therapies

Given that CGMs and questionnaires make it relatively easy to identify subjects at high risk for severe hypoglycemia, and some of the neuronal transmitters/circuits that contribute to HAAF have been identified, an unanswered call to action is the identification of potential pharmacological therapies that could improve awareness of hypoglycemia. The effects of various drugs on hypoglycemia awareness and counterregulatory responses have been assessed in preclinical models of HAAF, clinical models of inducible HAAF, and subjects with long-standing T1D and HAAF (Summarized in Table 2).

**TABLE 2:** Pharmacological therapies for impaired awareness of hypoglycemia (IAH).

Pharmacological therapies					
Drug	Model/Subject tested	Site of action	Mechanism of action	Effects on IAH	Citations
Miglitol	Rat	Gastrointestinal Tract	SGLT3 agonist, alpha-glucosidase inhibitor	Improvements in CRR, HYPO awareness not assessed	Jokiaho et al. (2022)
Carvedilol	Rat	CNS	blocks alpha-1, beta 1 and 2- adrenergic receptors	Improved both CRR and HYPO awareness via improved regulatory hormones and hunger	Farhat et al. (2019)
Modafinil	Mice and Humans	Brain (Hypothalamus and Tuberomammillary Nucleus)	Stimulates secretion of orexin neuropeptides/histamine and is a weak dopamine reuptake inhibitor	Mice - Improved HYPO awareness via activating orexin neurons and behavioral testing	Smith et al. (2004), Klement et al. (2014), Patel et al. (2022)
				Humans—increased autonomic symptom scores	
Formoterol	Rat	Brain (Ventromedial Hypothalamus) and Smooth Muscle	Beta-2-adrenergic receptor agonist	Improved epinephrine responses, awareness not assessed	Szepietowska et al. (2013)
Metoclopramide	Rat	Brain (chemoreceptor trigger zone in area postrema)	Dopamine receptor antagonist	Improved both CRR and HYPO awareness via improved counterregulatory hormones and food intake	Vieira De Abreu et al. (2018), Devore et al. (2022)
Exenatide	Human	Pancreas, hypothalamus, enteric nervous system	GLP-1 receptor agonist	Did not improve CRR or awareness	van Meijel et al. (2019)
Dapagliflozin	Human	Kidneys and Small Intestine (S1 and S2)	Inhibits SGLT2 and blocks reabsorption of glucose	Improves glucagon secretion, CGM metrics, and exogenous glucose needed during HYPO clamp, but no differences in symptom scores	van Meijel et al. (2021), Boeder et al. (2022), Urakami et al. (2023)
Losartan	Human	Adrenal Gland, Heart, and Brain	Angiotensin II receptor blocker	Lowers CRR responses and HYPO awareness	Deininger et al. (2001)
Amitriptyline	Human	Brain	Tricyclic antidepressant, serotonin reuptake inhibitor (alpha-adrenergic and histamine receptor)	Case study where drug cessation restored symptoms to hypoglycemia	Sherman and Bornemann (1988)
Imipramine	Human	Brain	Tricyclic antidepressant antagonist of acetylcholine receptors	Case study where a nondiabetic man had low glucose and no adrenergic symptoms to hypoglycemia with drug treatment	Shrivastava and Edwards (1983)
Selective Serotonin Reuptake Inhibitors (SSRIs)—Fluoxetine, Sertraline, Paroxetine	Human	Brain	Selective Serotonin Reuptake Inhibitors	Induced IAH in T1D subjects that had intact awareness prior to starting medications	Sawka et al. (2001)
Theophylline	Human	Pulmonary system and other smooth muscles/organs	Nonselective phosphodiesterase inhibitor and adenosine receptor antagonist	Improved CRR responses, induced sweating (symptom of HYPO), and decreased blood flow to the brain	de Galan et al. (2002)
Terbutaline	Human	Pulmonary system and other smooth muscles/organs	Selective Beta-2-agonist	Decreased nocturnal hypoglycemia, awareness not assessed	Cooperberg et al. (2008)
Naloxone	Human	Brain	Opioid receptor antagonist	Ameliorates epinephrine response and restores endogenous glucose production	Vele et al. (2011)

Abbreviations: impaired awareness of hypoglycemia (IAH), sodium dependent glucose transporter 3 (SGLT3), counterregulatory response (CRR), hypoglycemia (HYPO), central nervous system (CNS), glucagon-like peptide 1 (GLP-1), proximal tubule segment 1 and 2 (S1, S2), continuous glucose monitor (CGM), sodium dependent glucose transporter 2 and 3(SLGT2 and SGLT3), type 1 diabetes (T1D).

#### Animal studies

With the goal of augmenting the response to hypoglycemia, pharmacological interventions have targeted sites of action that are responsible for blood glucose sensing. When blood glucose falls, neurons in the brain (Thorens, 2012) and the periphery (Fournel et al., 2016) coordinate a counterregulatory response. One peripheral glucose sensor that responds to hypoglycemia lies within the portal-mesenteric vein (PMV) (Matveyenko et al., 2007) and signals the lateral hypothalamus and the paraventricular nucleus via the nucleus of the solitary tract (NTS) (Adachi et al., 1984). Recent studies suggest that PMV glucose sensing may be mediated via sodium-dependent glucose transporter 3 (SGLT3) receptors. Following antecedent hypoglycemia, miglitol (Glyset^©^, Pfizer, New York, NY, United States) a SGLT3 agonist, was shown to restore the counterregulatory response to hypoglycemia in rats (Jokiaho et al., 2022). Interestingly, authors concluded that miglitol could be used as a “day-after pill” restoring the counterregulatory response to avoid another incidence of hypoglycemia (Jokiaho et al., 2022).

The predominant glucose-sensing apparatus lies within the brain. Early studies identified the ventromedial hypothalamus (VMH) as a key glucose-sensing region (Borg et al., 1997; Routh, 2010; Chan and Sherwin, 2013), but several areas of the brain have been identified as having a key role in glucose counterregulation as part of an afferent and efferent neural circuit (Ritter et al., 1998; Beall et al., 2012).

In terms of testing responses to drug therapy, one study examined the effects of systemic and central (VMH) administration of a beta 2-adrenergic receptor agonist, formoterol, on the counterregulatory responses following hypoglycemia (Szepietowska et al., 2013). Systemic administration improved the glucose infusion rate and hepatic glucose production response to hypoglycemia; however, counterregulatory hormones did not change with formoterol administration (Szepietowska et al., 2013). While formoterol and miglitol improved counterregulation and hepatic glucose production of HAAF, awareness was not assessed in those studies and the effects of those drugs on IAH remain unknown.

In rodent models of HAAF, recurrent hypoglycemia consistently blunts the sympathoadrenal response (noted by a blunted plasma catecholamine response) (Powell et al., 1993). Unfortunately, the ability to determine hypoglycemia unawareness induced by recurrent hypoglycemia has been understandably more difficult to quantify in animal models (Sankar et al., 2020).

Of note, Farhat et al. (2019), targeted the VMH and assessed the preservation of the awareness of hypoglycemia using a non-selective β-adrenergic antagonist, carvedilol (Farhat et al., 2019). As model of IAH, recurrent antecedent treatment with 2-deoxyglucose (2DG) blunted the food intake response to insulin-induced hypoglycemia; yet rodents treated with carvedilol did not develop IAH (*i.e*., did not exhibit a blunted food intake response to hypoglycemia) (Farhat et al., 2019).

Another area of the brain that has been implicated in glucose sensing is the perifornical hypothalamus (PFH). Researchers focused on the orexin-glucose-inhibited neurons in the PFH (responsible for arousal) as a target for IAH and explored treatment with the anti-narcolepsy drug, modafinil (Teva Pharmaceutical Industries Ltd., United States) (Patel et al., 2022). Mice underwent a conditioned place preference test (surrogate test for IAH) prior to recurrent hypoglycemia and treatment. Compared to saline-treated mice, modafinil-treated mice adjusted their preference for the food-associated chamber after insulin-induced hypoglycemia. Additionally, researchers showed that modafinil restored glucose sensing by the orexin-glucose-inhibited neurons in the PFH (Patel et al., 2022). Modafinil is a dopamine reuptake inhibitor thus, it appears that dopamine signaling is potentially involved in the development of IAH. Consistent with this notion, metoclopramide (Teva Pharmaceutical Industries Ltd., United States), a dopamine (D2), receptor antagonist, was shown to improve both hypoglycemia awareness and counterregulatory hormone responses in response to insulin-induced hypoglycemia (Vieira De Abreu et al., 2018; Devore et al., 2022). Based on these preclinical results, the potential of this drug to restore awareness of hypoglycemia in subjects with T1D and IAH has advanced to a Phase 2 clinical trial (NCT03970720). Translation of these pre-clinical results to clinical trials remains an important step to validate potential drug therapies for the treatment of IAH.

#### Human studies

Drugs that work within the adrenergic system seem like an obvious target that might improve both the counterregulatory response and awareness of hypoglycemia (Cooperberg et al., 2008). Consistent with preclinical studies (Li et al., 2020), clinical studies have demonstrated that repetitive activation of the adrenergic system appears to contribute to hypoglycemia associated autonomic failure (Ramanathan and Cryer, 2011; Lontchi-Yimagou et al., 2020). Thus, some degree of adrenergic blockage within the CNS may serve to improve hypoglycemia awareness and hypoglycemic counterregulation, at least based on preclinical studies (Farhat et al., 2019; Farhat et al., 2021).

Another, similar pharmacological approach to treating IAH is targeting adenosine receptors to increase alertness and enhanced secretion of the counterregulatory hormones (De Galan et al., 2006). One study used theophylline, an adenosine-receptor antagonist, to determine its effects on IAH (de Galan et al., 2002). In response to hypoglycemia, subjects with diabetes and IAH treated with theophylline demonstrated an improved counterregulatory hormone response but theophylline did not improve hypoglycemia symptom scores (de Galan et al., 2002). However, another methylxanthine, caffeine, was shown to stimulate more symptomatic hypoglycemic episodes (i.e., improve awareness) (Watson et al., 2000).

The glucagon-like peptide-1 receptor agonist, exenatide, was used in a crossover trial in subjects with T1D and IAH (van Meijel et al., 2019). Subjects treated with exenatide for 4-week had no differences in frequency or time spent in hypoglycemia compared to the placebo group. Exenatide-treated subjects had similar symptom scores and counterregulatory hormone responses to that of the placebo group (van Meijel et al., 2019).

A sodium-glucose cotransporter-2 inhibitor, dapagliflozin, has shown effectiveness (van Meijel et al., 2021; Boeder et al., 2022; Urakami et al., 2023). Dapagliflozin treatment did not improve awareness of hypoglycemia, however, it did reduce the glucose infusion rates during the clamp (indicating an improvement in glucoregulatory response to hypoglycemia) (van Meijel et al., 2019). Using the same drug, another study assessed glucagon response in T1D subjects; however, subjects were on the lower end of the Clarke score (median 3, range 1–5), suggesting that awareness might have been present in some subjects. Similar to previous results, dapagliflozin treatment did not improve counterregulatory hormone responses, symptom scores, or recovery from hypoglycemia (Boeder et al., 2022).

Treatment with the CNS stimulant, modafinil, resulted in improved autonomic symptom scores, higher heart rates, higher glucagon concentrations during hypoglycemia, and improved scores on cognitive tests; however, the epinephrine response was not altered (Klement et al., 2014). Since modafinil was administered in non-diabetic subjects, IAH was not present (Klement et al., 2014). Conversely, another study also conducted in healthy subjects showed improvements in the norepinephrine response, but no other improvements in hormonal responses (epinephrine, growth hormone, and cortisol) or symptom scores during a hypoglycemic clamp (Smith et al., 2004). Both of these studies attribute the positive improvements seen in healthy subjects to γ-aminobutyric acid (GABA) signaling.

Modulating GABA signaling as a means to restore counterregulation and hypoglycemia awareness is supported by pre-clinical models (Chan et al., 2008). Clinically, antecedent GABA-A activation with the benzodiazepine, alprazolam, has been shown to blunt the neuroendocrine and autonomic nervous system responses to subsequent hypoglycemia in healthy humans (Hedrington et al., 2010). Consistent with these findings, antagonism of GABA with dehydroepiandrosterone (DHEA) can prevent the development of HAAF under experimental conditions in healthy humans (Mikeladze et al., 2016). Thus, with successful proof of concept studies in healthy humans, more recent studies in people with long-standing diabetes have shown that GABA administration significantly augmented the hormonal counterregulatory response to hypoglycemia (Espes et al., 2021).

The role of opioid receptors in the development and/or treatment of HAAF is an area of active investigation. Pre-treatment with opioid receptor agonists can impair the counterregulatory response to hypoglycemia (Carey et al., 2017). Conversely, pre-treatment with the opioid receptor antagonist (naltrexone) can prevent the development of an impaired counterregulatory response to hypoglycemia (Leu et al., 2009; Vele et al., 2011), but may not restore awareness of hypoglycemia in subjects with long-standing T1D and IAH (Moheet et al., 2015).

Based on animal studies that indicate a possible role for selective serotonin reuptake inhibitors (SSRIs) to augment the counterregulatory response to glucoprivation (Baudrie and Chaouloff, 1992), clinical studies have demonstrated that 6-week treatment with SSRIs augmented counterregulatory, but not symptom responses, to hypoglycemia in nondiabetic people (Briscoe et al., 2008a; Briscoe et al., 2008b). It remains to be determined if these beneficial effects of SSRIs are mediated by the inhibition of neuronal serotonin uptake or via inhibition of norepinephrine transport in the CNS (Chaouloff et al., 1991). It also remains to be determined why hypoglycemia awareness was not improved with SSRI therapy.

With a goal of improving both the counterregulatory response to hypoglycemia and awareness of hypoglycemia, the development of novel drugs and/or the repurposing of existing FDA approved drugs remains an important area of research.

## Summary and future directions

IAH continues to be a complication in people with both T1D and T2D who seek optimal glycemic control with insulin therapy. Providers who care for patients with diabetes should inquire about hypoglycemia and IAH with a view towards considering treatment options. This review shows that there are several advances in technology and educational approaches that can improve hypoglycemia awareness. With regards to pharmacological treatments, basic science research in animal models is continuing to elucidate the mechanism(s) responsible and these novel treatments for IAH are being advanced into clinical trials. Future studies should focus on these possible mechanisms to develop more targeted therapies for patients who suffer from IAH.
